# Quantitative Evaluation of the Severity of Acute Illness in Adult Patients with Tick-Borne Encephalitis

**DOI:** 10.1155/2014/841027

**Published:** 2014-05-07

**Authors:** Petra Bogovic, Mateja Logar, Tatjana Avsic-Zupanc, Franc Strle, Stanka Lotric-Furlan

**Affiliations:** ^1^Department of Infectious Diseases, University Medical Centre Ljubljana, Japljeva 2, 1525 Ljubljana, Slovenia; ^2^Medical Faculty, Institute for Microbiology and Immunology, Zaloška 4, 1000 Ljubljana, Slovenia

## Abstract

The aim of the present study was to quantify the severity of acute illness in patients with tick-borne encephalitis and to ascertain this approach by comparing it to standard clinical assessment. We designed scoring system for quantification of the severity of acute illness in patients with tick-borne encephalitis. Certain number of points was allotted to the presence, intensity, and duration of individual symptoms/signs. According to the obtained score the disease was classified as mild, moderate, and severe. Tick-borne encephalitis was assessed clinically as mild when only signs/symptoms of meningeal involvement were found, moderate in case of monofocal neurological signs and/or mild to moderate signs/symptoms of central nervous system dysfunction, and severe in patients with multifocal neurological signs and/or symptoms of severe dysfunction of central nervous system. By designed scoring system 282 adult patients, 146 males and 136 females, average aged 52.2 ± 15.5 years (range 15–82 years), with confirmed tick-borne encephalitis, were prospectively assessed. In 279/282 (98.9%) patients the severity according to clinical assessment matched with the score ranges for mild, moderate, and severe disease. The proposed approach enables precise and straightforward appraisal of the severity of acute illness and could be useful for comparison of findings within/between study groups.

## 1. Introduction

Tick-borne encephalitis (TBE) is one of the most important human central nervous system infections in several European and Asian countries. It is caused by RNA virus belonging to the genus* Flavivirus* of the family Flaviviridae. The genetic analysis shows the existence of three subtypes of TBE virus designated as Western European, Far-Eastern, and Siberian subtype [1].

TBE is endemic in a vast area ranging from Europe, through Siberia, and far-eastern Russia to northern China and Japan [2]. It is present in regions at least of 27 European countries. The highest incidence is registered in Latvia, Estonia, Slovenia, and Lithuania [3]. A substantial increase in TBE incidence was reported in the years between 1990 and 2007 in the majority of European countries endemic for TBE, and new risk areas are discovered every year. This increase may have been attributed to many different factors such as climatic, social, political, ecological, economical, and demographic changes [4–7].

TBE is a seasonal disease. In central Europe the large majority of cases occur from April to November, peaking in June and July, sometimes with secondary rise in September and October. The principal vector of the Western European TBE virus subtype (which is endemic in scattered areas within central, eastern and northern Europe) is the hard tick* Ixodes ricinus*. Human infections can also result from the consumption of infected unpasteurised dairy products although these occur less frequently [3, 6, 8].

As with many arboviruses, the majority of TBE virus infections are asymptomatic [3, 8–10]. In approximately two-thirds of patients who develop central nervous system involvement the disease caused by Western European virus subtype has a characteristic biphasic course. The initial, viremic phase usually begins 7–14 days after a tick bite and manifests with nonspecific symptoms such as headache, fatigue, myalgias, and moderate fever. This phase persists for 1–8 days and is followed by an improvement or even an asymptomatic interval of about 1-week duration. Then the second febrile phase develops and presents as meningitis in about 50% of adult patients, as meningoencephalitis in about 40%, and as meningoencephalomyelitis in about 10% [3]. Meningitis is usually accompanied by high fever, headache, nausea, and vomiting. Signs of meningeal irritation usually occur but may not be pronounced. Encephalitis is characterized by a disturbance of consciousness ranging from somnolence to stupor and, in rare cases, coma. Other symptoms include restlessness, tremor of extremities, and fasciculations of the tongue, vertigo, and cognitive function disturbance. Meningoencephalomyelitis is the most severe form of the disease. It is characterized by flaccid paresis that usually develops during the febrile phase of the illness. The upper extremities are affected more frequently than the lower ones and the proximal segments more often than the distal ones. The case-fatality rate of the disease in central Europe is 0.5–2% [3, 8].

As there is no antiviral treatment for TBE available, the only effective mode of prevention is active immunization. The efficacy of the vaccine is high (98–99.5% after complete basic vaccination) with no significant differences between age groups [11, 12].

It has been known that the severity of the disease varies between different study populations as well as between different geographical regions. According to published information TBE caused by Far-Eastern virus subtype has a more severe clinical course than that caused by other TBE virus variants [3]. In central Europe TBE is milder in children than in adults and is especially severe in those above the age of 60 years [3, 13–15]. However, a severe course of TBE in children with residual symptoms, including neuropsychological impairment, and even death, has been reported [16].

The approaches used for the clinical assessment of the severity of TBE as reported in the literature are heterogeneous and rather imprecise [1, 3, 8] and therefore do not permit accurate comparison within and between (study) groups of patients with TBE. This was a stimulus for quantification of symptoms and signs of acute illness in patients with TBE using a standardized questionnaire.

## 2. Patients and Methods

### 2.1. Patients

This prospective clinical study was carried out at the Department of Infectious Diseases, University Medical Centre Ljubljana, Slovenia, in years 2005 and 2006. We included all patients aged 15 or older with confirmed TBE hospitalized at our department in these two years. Informed consent was obtained from all the participants.

A confirmed case of TBE was defined as febrile patient with clinical signs and/or symptoms of meningitis or meningoencephalitis, an elevated cerebrospinal fluid cell count (>5 × 10^6^ cells/L), and the presence of serum IgM and IgG antibodies to TBE virus. The presence of IgG antibodies without IgM was interpreted as evidence of previous TBE virus infection or vaccination against TBE. The presence of serum IgM and IgG antibodies against TBE virus was detected with Enzygnost Anti-TBE Virus (IgM, IgG) test (Dade Behring Marburg GmbH, Marburg, Germany) performed according to the manufacturer's instructions.

At initial examination on the day of hospital admission a detailed epidemiological and medical history was obtained, physical examination was performed, and routine blood and cerebrospinal fluid tests were done including cell counts, concentration of total proteins, glucose, albumin, IgG, IgA, and IgM. During hospitalization the presence of signs and symptoms of TBE was evaluated and registered on daily basis.

### 2.2. Methods

TBE was assessed clinically as mild when only signs or symptoms of meningeal involvement were found, moderate in case of monofocal neurological signs and/or mild to moderate signs or symptoms of central nervous system disfunction, and severe in patients with multifocal neurological signs and/or signs or symptoms of severe dysfunction of central nervous system [17].

For the quantitative evaluation of severity of the disease a standardised questionnaire, summarised on Table 1, was used. A certain number of points (from 1 to 9) were assigned for the presence, intensity, and duration of each individual symptom or sign including the presence and duration of headache, fever, vomiting, and meningeal signs; the presence of tremor, pareses, urine retention, and cognitive function disturbances; the presence and intensity of conscious disturbances; and the need for and the duration of treatment of elevated intracranial pressure. The absence of particular symptom/sign was given zero points. The score system had been formerly calibrated on a smaller group of patients. These data, obtained prior to the present study, had suggested that score ranges 0–8, 9–22, and >22 might correspond to clinically mild, moderate, and severe disease, respectively.

The findings obtained by the new scoring system were compared with those acquired independently by clinical evaluation performed by experienced clinicians who were not aware of the scoring system results.

### 2.3. Statistical Analysis

Epi Info software, version 3.3.2 (Centers for Disease Control and Prevention, Atlanta, USA), was used for data processing and statistical analysis. Differences in the quantitative data were analyzed by the Kruskal-Wallis test and differences in categorical data by Yates's corrected *χ* ^2^ test or Fisher's exact test. Two-tailed *P* values of <0.05 were interpreted as statistically significant.

## 3. Results

To test this newly designed scoring system 282 consecutive adult patients with confirmed TBE who were hospitalized at our department in 2005 and 2006 were prospectively assessed. There were 146 (51.8%) males and 136 (48.2%) females, with average age 52.2 ± 15.5 years (range, 15–82 years).

**Figure 1:**
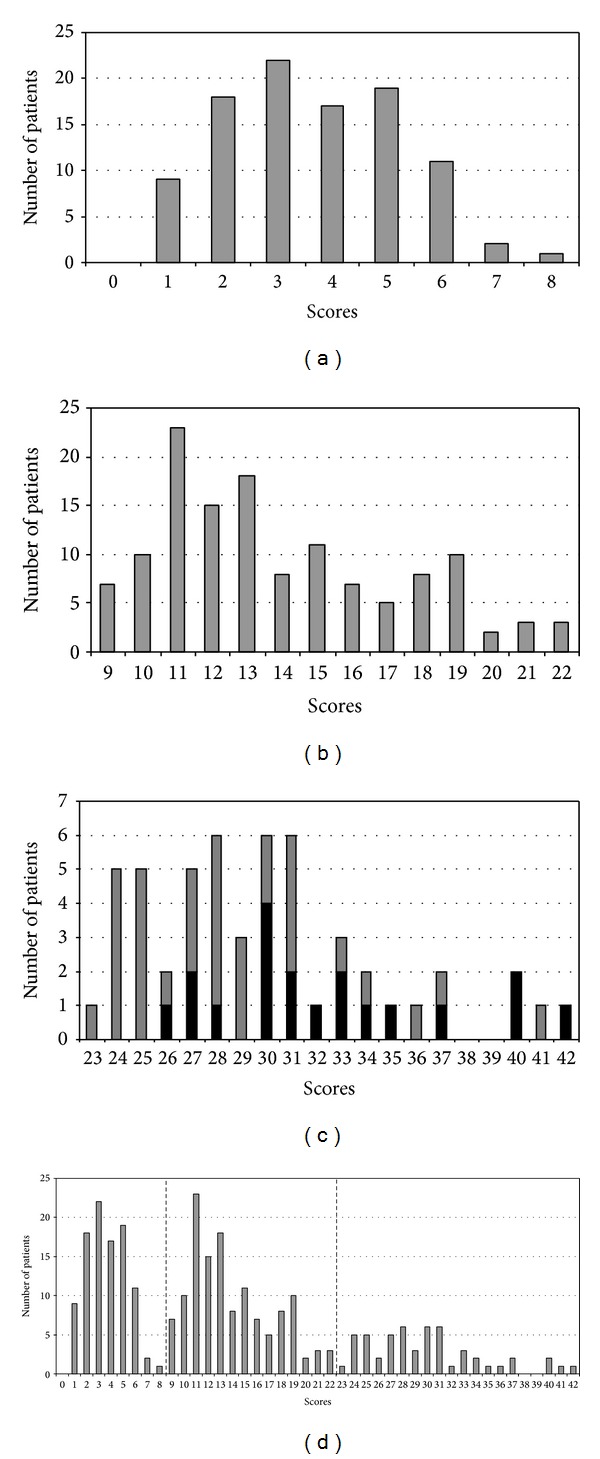
Distribution of scores within the group of patients with mild, moderate, and severe disease. (a) Distribution of scores in a group of 99 patients with mild disease; median score 4, range 1 to 8; the two patients who were clinically classified as having an illness of moderate severity acquired score 6. (b) Distribution of scores in group of 130 patients with moderately severe disease; median score 13, range 9 to 22; the patient who was clinically classified as having severe illness got score 21. (c) Distribution of scores in group of 53 patients with severe disease; median score 29, range 23 to 42; of 19 patients treated in the ICU (shown in black bars) three required artificial processes of ventilation; these patients acquired scores 27, 31, and 40; two patients died. (d) Summary distribution of scores in patients with TBE, assessment of the severity of acute illness: scores ≤8 mild illness; scores 9–22 moderately severe illness; scores ≥23 severe illness.

In 279 (98.9%) out of 282 patients severity according to clinical assessment matched the score ranges for mild (0 to 8 points), moderate (9 to 22 points), and severe (>22 points) disease (Table 2). In 3 (1.1%) out of 282 patients alignment according to clinical evaluation and according to quantitative evaluation by scores of questionnaire did not match. In all three cases of mismatch questionnaire favoured milder disease. Patients with severe illness who required treatment in the intensive care unit acquired a score of at least 26. Two patients died. Distribution of scores within the group of patients with mild, moderate, and severe disease is shown in Figure 1.

## 4. Discussion

TBE is an endemic disease in a large part of Slovenia. It has been known to be present in our country since 1953 [18], and its notification has been mandatory since 1977. During the last 5 years 166–304 TBE cases were registered annually, and the annual incidence ranged from 8.1 to 14.9 cases/100.000 inhabitants. Despite the high incidence rate of the disease and several severe cases the usage of vaccine against TBE in Slovenia is low and is predominantly restricted to persons at professional risk [11, 19].

More than half of the registered patients with TBE in Slovenia are hospitalized at our department, which means that as a rule we take care of more than 100 adult patients with TBE per year, the large majority in the 6-month period between May and October. Pronounced variations in the numbers of hospitalized patients comparing individual years are observed and there is an impression that during some seasons the course of acute disease is more severe than during others. However, the approaches currently used for the clinical assessment of the severity of TBE are imprecise and often inconsistent and therefore do not permit accurate comparison within and between groups of patients with TBE. This prompted us to quantify the symptoms and signs of acute illness in patients with TBE using a standardized questionnaire. Using this questionnaire number of points between 1 and 9 (Table 1) was allotted to the presence, intensity, and duration of each individual symptom or sign. The score system which had been formerly calibrated on a small group of patients was tested in the present study on 282 consecutive adult patients with confirmed TBE who were hospitalized at our department from 2005 to 2006. The findings obtained by the new scoring system were validated by comparison to those acquired independently by a currently used clinical evaluation. The evaluation was performed by experienced clinicians who were not aware of the scoring system results and who interpreted the disease as mild when only signs or symptoms of meningeal involvement were found, moderate in case of monofocal neurological signs and/or mild to moderate signs or symptoms of central nervous system disfunction, and severe in patients with multifocal neurological signs and/or signs or symptoms of severe dysfunction of central nervous system.

The findings of the two approaches were highly congruent. In only 3 (1.1%) out of 282 patients, alignment into the groups according to quantitative evaluation by scores did not match the categorisation according to clinical evaluation. In all three cases of mismatch questionnaire favoured milder disease and the scores obtained were very near to the upper score limit for the corresponding group. Thus, comparing the grouping into three basic severity categories, the quantitative approach was in very good correlation with the classical clinical assessment findings.

The advantage of the quantitative approach is that it enables not only classification into three basic severity categories but also differentiation within individual (mild, moderately severe, and severe) illness group. While the scores within the group with mild and moderately severe disease showed a bell-shaped curve distribution, the distribution of scores in patients with severe acute illness was more widely spread (Figures 1(a)–1(d)).

We would like to stress that the findings were obtained in adult patients and the scoring system may not be valid for children with TBE who are younger than 15 years and not yet evaluated with this system. The usefulness of the approach may also be limited for differentiation within the subgroup of patients with the most severe disease who need treatment in the intensive care unit.

We believe that the proposed quantitative approach enables a reliable, precise, and straightforward appraisal of the severity of acute TBE in individual patient and thus could be useful for comparison of the severity of acute illness within and between groups of adult patients with TBE.

## Figures and Tables

**Table 1:** Questionnaire for the evaluation of the severity of acute illness in patients with tick-borne encephalitis.

Present symptom/sign	Duration (days)	Points^a^
Headache	1–5	1
	>5	2
Fever^b^	1–3	1
	4–7	2
	>7	3
Vomiting	1–3	1
	>3	2
Meningeal signs	1–5	1
	>5	2
Tremor	/	7
Pareses		
1-2 extremities	/	7
>2 extremities and/or trunk	/	9
Urine retention	/	5
Cognitive function disturbance	/	5
Conscious disturbance		
Somnolence	/	3
Stupor/coma	/	7
Therapy for elevated intracranial pressure^c^	1–5 doses	3
	>5 doses	5

/ Not applicable.

^a^The absence of an individual symptom/sign was given zero points.

^b^>37.5°C.

^c^250 mL 20% manitol followed by 20 mg furosemide parenterally (up to two times daily).

**Table 2:** Correlation of patients with tick-borne encephalitis according to score and clinical evaluation.

Severity of illness	Alignment according to score	Alignment according to clinical evaluation	*P* value
Mild	99 (35.1)	97 (34.4)	0.93
Moderate	130 (46.1)	131 (46.5)	1.0
Severe	53 (18.8)	54 (19.1)	1.0

Data are number (%) of patients.
